# Symptoms from Rhus Poisonings*Read before the Kansas State Homœopathic Medical Society, May, 1895.

**Published:** 1895-08

**Authors:** Robert A. Billings

**Affiliations:** Kansas City, Kansas


### SYMPTOMS FROM RHUS POISONINGS.*

Robert A. Billings, M. D., Kansas City, Kansas.

Case I.—Miss B., aged nine years, while visiting in Central Kansas City last August, went to the timber to play with some other children, and they got into some poison sumach; Miss B., after going to bed that night, began to complain to her mother of an itching, burning, crawling sensation all over her body, and could not sleep or have any cover on her, and kept her mother awake all night. Her mother found, on examining her next morning, that she was broken out all over her body with a smooth red rash. In a few hours the rash disappeared and she began to break out in different places all over the body with large urticaria-like blotches; some as large as the palm of the hand, and some much larger, with large watery blisters surmounting their tops. This condition lasted four days. She was unable to rest or sleep either day or night on account of the severe burning, itching pains. On the fourth day she began to have chills. Chills followed by fever, chills or cold all day; fever all night; so cold could not get enough cover or clothes on to get her warm. When she would get near the stove to get warm it would aggravate her symptoms so that she could not endure it. These symptoms continued. On the seventh or eighth day she began to get drowsy; drowsy all the time. On the twelfth day, the above symptoms all continuing, her feet, toes, hands, and fingers began to swell. Her feet and toes swelled so badly that she could not get on her shoes. Toes and heels hurt so badly she could hardly step on them. Hands and fingers swelled so badly she could not get on her gloves or bend her fingers. Pains in her feet, toes, hands, and fingers; pains all over. Severe neuralgic pains. Rest and warmth in the house aggravated; exercise and cool air ameliorated. During all this time her appetite was good, her bowels regular, except she had diarrhœa for three days.

---

* Read before the Kansas State Homœopathic Medical Society, May, 1895.

Her mother had got very much worried about her by this time. In spite of salt and soda baths and all the other remedies she and her friends could think of, the girl got gradually worse; she cut her visit short and returned to the city, where she could have the girl under my care. It took me three weeks to antidote the poison. I finally did so with Anacard-orient.

Case II.—While walking along a creek in Western Kansas one damp morning, I passed through some poison sumach. A friend who was with me remarked that I would most likely get poisoned as I had on shoes. After being in bed a short time that evening an itching, burning, crawling sensation began on my legs, just about where my shoe tops came to. It kept gradually getting higher on my legs, and I thought there were about four thousand fleas crawling up my legs all at the same time. "By the way," I was out in that part of Kansas where fleas are plenty. I did not sleep much that night. I tried all sides of the bed, and got up several times to get away from the fleas, as I thought my tormentor was, but it did no good. On examining my limbs in the morning I found them covered with a smooth rosy-red rash, as high up as the groin. I now knew what was the cause of my having such a bad night. I had got a dose of poison sumach. At noon that day when I got out of my buggy for dinner I found I was so lame that at first I could hardly walk, but as I got to moving around some I soon limbered up so I could walk very well. The lameness was all in the adductor muscles of the thighs. On getting out of my buggy in the evening I found that I was much lamer than at noon. It was some time before I could take a step, I was so lame, and it hurt me so to use the adductor muscles of the thighs that I thought I would have to give up the attempt to walk that evening. But on moving around a little I soon found that I could walk very well after some exercise. The itching, burning, crawling sensation began to get worse than it was the night before, although it had not bothered me much during the day. On examining myself I found that I was broken out all over my body and limbs, except my face, hands, and feet, with a smooth red rash, looking very much like the eruption of measles when fully out, and running together, forming irregular-shaped plaques. I did not take off my clothes and go to bed that night. I walked all night. When I would sit down for a few minutes the severe burning, itching pains would cause me to move on again, and as soon as I remained still for a few minutes I would get so stiff that I could hardly move again; so I kept moving all night. It was the only way that I could get any ease. By morning I had had enough of the symptoms of Rhus-tox poison to suit me. I began to take Apis-mell.$^{1x}$, and by evening of that day I was better. A strong salt water bath on going to bed and I was able to get some sleep. By the next night I was all right. This is the first time I have ever been poisoned by the poison sumach, and I hope it will be the last.

I have been poisoned many times by the poison ivy or vine, Rhus Radicans, and have seen many cases of poisoning by it. The effects of the poison are quite different from that of the poison sumach. If the poison of the poison vine comes in contact with the leg, it produces an intense inflammation of the skin for a few inches around. The skin swells greatly, is very red with intense burning, itching pain; soon large watery blisters, or a fine vesicular rash breaks out on the inflamed area, then the inflammation subsides and the part affected returns to its normal condition. No difference what part of the body the poison comes in contact with, it stays confined to that region; it doesn't seem to have any inclination to spread far.

The effect of the poison sumach, Rhus-tox., is far deeper. It makes no difference what part of the body the poison comes in contact with, it has a tendency to spread over large surfaces, and attack deeper tissues, and goes on for months or years if not stopped by proper treatment.

The symptomatic indications for Rhus-tox. are found in the continued fevers, the eruptive fevers and rheumatisms.

For Rhus Radicans in the local inflammations.

Of the antidotes to the Rhus poisons, Apis-mell. is a good one in the acute stage, but of no use later on. It should be given in the 1x or Ø.

Bryonia is good in some cases. Croton-tig. is the remedy where there is a fine vesicular rash or small boils.

Anacardium-orient. is the remedy where there are large watery blisters on an inflamed base, with swelling of the various joints.

Belladonna is mentioned by some as a good antidote but I have not found it of much use.

